# The usefulness of immunohistochemistry in tissue microarrays of Human Papillomavirus negative adenocarcinoma of the uterine cervix

**DOI:** 10.1186/1756-0500-3-54

**Published:** 2010-03-03

**Authors:** Michael Odida, Belen Lloveras, Nuria Guimera, Elisabete Weiderpass

**Affiliations:** 1 Department of Medical Epidemiology and Biostatistics, Karolinska Institutet, 171 77, Stockholm, Sweden; 2 Department of Pathology, Faculty of Medicine, Makerere University, PO Box 7072, Kampala, Uganda; 3 Unit of Infections and Cancer, Cancer Epidemiology Research Programme, Institut, Català d' Oncologia, Av Gran via s/n km 2,7, 08907 L'Hospitalet de Llobregat, Spain; 4 Department of Pathology, Hospital del Mar-IMAS Pg Maritim 23, 08004, Barcelona, Spain; 5 Department of Etiological Research, Cancer Registry of Norway, Pb 5313, Majorstuen, 0304 Oslo, Norway; 6 Department of Community Medicine, Tromso University, 9037 Tromso, Norway; 7 Department of Genetic Epidemiology, Folkhälsan Research Center, PB 211, 00250, Helsinki, Finland

## Abstract

**Background:**

The origin of adenocarcinomas presenting on the cervix uteri may be doubtful, i.e. whether it is of cervical or endometrial origin, due to the overlapping morphological features. In HPV negative samples, further tests may be needed to ascertain the nature of the tumours. We aimed to explore the use of immunohistochemistry profiles in tissue microarrays in archived samples of adenocarcinoma (ADC) of the cervix from Uganda that tested negative for HPV DNA.

**Findings:**

Five commercially available antibodies were tested in tissue array sections immunostained utilizing the avidin-biotin (AB) technique. In 26 ADC samples, HPV was detected in 13, p16 in 15 (8 in HPV positive and 7 in HPV negative), CEA in 12, vimentin in 6, ER in 0, and PR in 2. Among the 13/25 HPV negative ADC samples, five were positive for CEA suggesting endocervical origin, and three were vimentin positive (one had a mucinous endocervical histological pattern and two were ADC, not otherwise specified, most likely of endometrial origin).

**Conclusions:**

The immunoprofiles of ADC with the antibodies studied are rather nonspecific. By using immunohistochemistry in 13 HPV negative ADC, endocervical tumour origin was suspected in five CEA positive cases while two out of three vimentin positive samples were probably of endometrial origin, suggesting that CEA and vimentin may be valuable in distinguishing HPV negative cervical adenocarcinomas from endometrial adenocarcinomas.

## Background

Although the role of HPV in cervical adenocarcinoma appears well established [1-3], the nature of adenocarcinoma arising from the cervix may be difficult to define due to the overlapping morphological features between cervical adenocarcinomas and endometrial adenocarcinomas. While endocervical adenocarcinomas express p16 and CEA [4] and whereas endometrial adenocarcinomas usually showed vimentin and hormonal receptors [5], the results may be inconclusive. However, CEA has been reported to be expressed in both cervical and endometrial adenocarcinomas [6], while the expression of vimentin was noted to be weak and focal in many endometrial adenocarcinomas [5]. An alternative view is that some expression of these markers may reflect differentiation (mucinous versus endometrioid) compared to the histogenetic site of origin (endometrial versus cervical) [7]. In order to resolve the problem, HPV testing has been used in attempts to distinguish these tumours [8]. Extending this approach, a recent study evaluated the use of HPV DNA, p16 and hormonal status to determine the origin of cervical tumours [9].

HPV negative adenocarcinomas in the same site may originate either from cervical or endometrial cells. We postulated that immunohistochemistry could be of use in HPV negative cervical tumours from Uganda.

We aimed to explore the utility of immunohistochemistry profiles, i.e. the expression of p16, carcinoembryonic antigen (CEA), vimentin, estrogen receptor alpha (ER) and progesterone receptor (PR), in tissue microarrays in archived samples of ADC that had been tested for HPV DNA, and in particular to assess if it would be possible to define the tissue of origin of HPV negative adenocarcinoma samples. This was based on the use of CEA as a marker of endocervical ADC, vimentin and hormone receptors (markers more related to endometrial ADC) and p16 as a proxy marker for HPV infection. p16 is a protein encoded by p16INK4a gene and has been used as an indirect assay for HPV infection [10]. CEA is an onco-fetal protein which has been touted as a useful antibody in distinguishing between endocervical adenocarcinoma and endometrial adenocarcinoma [6]. Vimentin is characteristically positive in endometrial adenocarcinoma [5], although positivity has also been reported in cervical adenocarcinomas [6]. ER and PR are usually positive in endometrial adenocarcinoma and negative in endocervical adenocarcinoma [4]. They are used usually to exclude endocervical origin of tumours.

## Materials and methods

Cervical carcinoma samples were retrieved from the archives of the Department of Pathology, Makerere University, Kampala, Uganda, and were diagnosed during the period 1968-1990. The study protocol has been approved of Higher Degrees Research & Ethics Committee at Makerere University, Uganda.

### HPV testing

The cervical carcinoma samples sections selected for HPV testing were digested with proteinase K and the resulting extract was used for PCR. SPF10 PCR was performed using 10 μl of a 1:10 dilution of the DNA extract in a final reaction volume of 50 μl. The amplified PCR products were tested using probe hybridization with a cocktail of conservative probes recognizing at least 54 mucosal HPV genotypes in a microtiter plate format for the detection of HPV DNA. Optical densities (OD450) were read on a microtiter plate reader. HPV DNA positive samples were subsequently analysed by HPV SPF10-LIPA25 (version 1: produced at Labo Biomedical Products, Rijswijk, The Netherlands) [10,11], a reverse hybridization technique that detects 25 high-risk and low-risk HPV types (6, 11, 16, 18, 31, 33, 34, 35, 39, 40, 42, 43, 44, 45, 51, 52, 53, 54, 56, 58, 59, 66, 68, 70, 74). The sequence variation within the SPF10 primers allows the recognition of these different HPV genotypes, except for the types 68 and 73 as their interprimer regions are identical and cannot be distinguished on this test. After PCR, 10 μl of the amplimers was used to perform reverse hybridization for HPV genotype identification. The positive hybridization on the strips is visualized as a purple band by means of a precipitating colour substrate on the probe site. All SPF10-LIPA25 PCR detection and typing was performed at the facilities of DDL Diagnostic Laboratories (DDL, Voorburg, Netherlands) and at Institut Català d'Oncologia (ICO, Barcelona). Detailed HPV results were published elsewhere [12].

### Tissue Microarrays (TMA)

Three punches (gauge 1 mm) of pre-existing paraffin embedded tissues were obtained from each block and then re-embedded in an arrayed master block using the manual tissue microarrayer (Beecher Instruments, Silver Spring MD). The punch specimens were performed in the most representative areas of the tumours, discarding necrosis or artefacts. Four micron paraffin sections were cut from the TMA blocks and deparaffinised through alcohols and xylene before immunostaining.

### Immunohistochemistry (IHC)

Monoclonal antibodies included: CEA (clone B0194-11M-P, BioGenex), p16 INK4a, p16 (clone JC8, Biocare Medical), Vimentin (clone V9, Dako), estrogen receptor alpha, ER (clone 1D5, Dako), progesterone receptor, PR (clone PgR 636, Dako).

Heat Induced Epitope Retrieval (HIER) was done using a pressure cooker as a heating device. Retrieval solutions that were used were: Citrate buffer for Vimentin, PR, ER; EDTA 10% for p16 and Saponin for CEA. Incubation was done at room temperature for 30 min for all the antibodies.

The Dako Autostainer universal staining system was used with the EnVision+ Dual Link System-HRP, a two-step IHC staining technique. This system is based on an HRP labelled polymer which is conjugated with secondary antibodies.

The final reaction was done with diaminobenzydine and slides were counterstained with hematoxylin. The immunohistochemical stains were interpreted by one of the authors (BL) and the results were reported as positive or negative using the following criteria for each antibody. ER and PR were considered positive when more than 10% of the tumour cells displayed nuclear positive staining. p16 was considered positive/overexpressed if more than 75% of the tumour cells showed cytoplasmic and/or nuclear staining, CEA was considered as positive if more than 10% of the neoplastic cells showed cytoplasmic positive staining, and vimentin was considered positive only when more than 20% tumour cells displayed cytoplasmic staining. The cut-offs for most antibodies were those used at laboratory in Barcelona, while the 75% for p16 was chosen because this cut-off is both sensitive and specific for HPV related cervical adenocarcinoma [13].

### Statistical analysis

The chi-square test was used to determine significant differences between HPV positive and HPV negative adenocarcinomas and expression of, p16, CEA, ER, PR and vimentin.

## Results

In total 26 samples were successfully analysed for HPV and immunohistochemistry. In these, HPV DNA was detected in 13/26 (50%) of ADC (Table 1). Overexpression of p16 was detected in 15/26 (57.7%) ADC. Of the 15 p16 positive cases, 8 (53.3%) were HPV positive and 7 (46.7%) were HPV negative. The reactivity was both cytoplasmic and nuclear in positive samples.

**Table 1:** HPV positivity and immunohistochemistry markers in cervical adenocarcinomas (ADC)

	Adenocarcinoma (ADC)
*HPV positivity*	48.1% (13/26)
*p16 *≥ 75%	57.7% (15/26)
*CEA *> 10%	48.0% (12/25)
*Vimentin *≥ 20%	23.1% (6/26)
*ER *≥ 10%	0.0% (0/26)
*PR *≥ 10%	7.7% (2/26)

**Figure 1:**
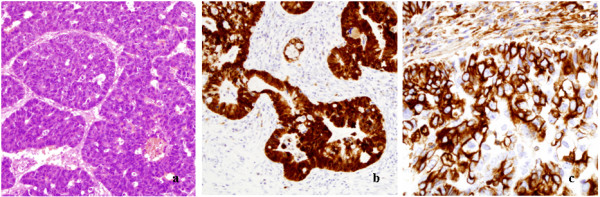
**Adenocarcinoma**. Hematoxylin-eosin (a), p16 positive staining (b) and vimentin positive staining (c).

CEA was positive in 12/25 (48%) ADC (Table 1). Of these, seven were HPV positive and five HPV negative. All five HPV negative and CEA positive had histological features of endocervical ADC, suggesting endocervical origin (Table 2). Vimentin was positive in 6/26 (23.1%) ADC, three of them being HPV positive. Of the three HPV negative cases, one had a mucinous endocervical histological pattern and two were ADC not otherwise specified (NOS), most likely of endometrial origin. ER was not detected in any sample and PR were positive in two samples, both HPV positive (Tables 2 and 3). Seven ADC samples were negative for all the three markers, of these two were HPV positive. Among the other five samples, two had endocervical histological features, two were serous, and one was ADC NOS. (Table 2). The positivity for each of the immunohistochemistry markers did not differ in HPV positive and negative ADC (Table 3, Figure 1).

**Table 2:** Immunohistochemistry patterns by HPV positivity in archival adenocarcinoma samples from Uganda*

HPV positive (N = 12/25)				HPV negative (N = 13/25)		
***CEA***	***Vimentin***	***ER***	***PR***	**% with this pattern**	**% with this pattern**	**Histological subtype**

+	-	-	-	58.3% (7/12)	38.5% (5/13)	Mucinous Intestinal (2) Mucinous Endocervical (1) Mucinous NOS (1) NOS (1)
-	+	-	+	16.7% (2/12)	0.0% (0/13)	-
-	+	-	-	8.3% (1/12)	23.1% (3/13)	Mucinous Endocervical (1) NOS (2; probable endometrial)^†^
-	-	-	-	16.7% (2/12)	38.5% (5/13)	Mucinous Endocervical (1) Mucinous Villoglandular (1) NOS (1)^†,§^ Serous (2; probable endometrial)^†^

*Out of the 26 samples 25 were stained with all the antibodies and HPV tested

^†^After immunohistochemistry and histological subtype diagnosis an endometrial origin cannot be discarded

^§^No further classification because of lack of markers

**Table 3:** Immunohistochemical stains by histological subtype of cervical adenocarcinoma*

	HPV +	HPV-	p value
***p16 (+)***	8/12	7/13	0.69
***CEA (+)***	7/12	5/13	0.53
***Vimentin (+)***	3/12	3/13	1.0
***PR (+)***	2/12	0/13	0.22
***ER (+)***	0/12	0/13	-

*Out of the 26 cases, histological sections stained with all the antibodies were available in 25

## Discussion

Histological subtypes of endocervical and endometrial ADC have some overlapping features, and poorly differentiated tumours or small biopsies are difficult to classify.

The differential diagnosis between endocervical and endometrial ADC is a common problem in surgical pathology, that can be approached using different immunohistochemical antibodies, the most accepted panel markers being CEA (as a marker of endocervical ADC), vimentin and hormone receptors (markers more related to endometrial ADC). HPV DNA detection by molecular techniques has proved valuable [8]. However in our series we found a much higher percentage of HPV negative ADC (50%) than we expected.

As a comparison, 85% of samples were positive in the whole series of the RIS TT study, which used similar HPV testing methods [14]. This could probably be explained by poor sample processing conditions in Uganda, including problems in sample transport, type of fixative (inconsistent use of buffered or non-buffered formalin) and duration of fixation, leading to poor DNA and protein preservation and consequently immunohistochemistry and PCR with equivocal results.

Overexpression of p16 has been used as a surrogate marker of high risk HPV infection [10]. Only 57.7% of our ADC samples were p16 positive; considering the probable rate of false negative of 15% (the rate of false negative in SCC) we should have about at least 25% (i.e. 57.7% + 15% = about 75%; 100% - 75% = 25%) of p16 negative ADC samples that remain unexplained. Being a usual problem in diagnosis, the possibility of endometrial cancer has to be considered. Moreover, p16 itself cannot be considered an ideal marker of HPV infection in ADC, since many samples of HPV negative cervical ADC have also been found to express p16 [15]. This raises the possibility of HPV-independent mechanisms of p16 overexpression in some cervical ADC, similar to what has been observed in Bowen's disease [16]. Besides, some endometrial cancer can also display overexpression of this marker [5]. Among the 13 HPV negative ADC, there were some features that support endocervical origin, i.e. CEA expression in five cases and the histological features were in favour of endocervix in three others although IHC results were inconclusive. Other studies have also found that CEA is usually positive in cervical ADC, although some samples may be negative [4,17,18]. Three of our samples showed vimentin expression which is usually related to endometrial origin [5], however one of them displayed a morphological pattern suggestive of endocervical differentiation. The expression of vimentin in cervical ADC has been reported previously [6]. The absence of ER and low expression of PR is not surprising, considering that few samples of cervical ADC are usually ER or PR positive [4].

Thus, endocervical origin seems confirmed in some samples, endometrial cancer were suspected in four others (two vimentin positive and two without conclusive IHC results but were serous ADC) and one could not be further classified due to lack of histological or IHC markers.

In view of overlapping and diverge morphologic heterogeneity of both cervical and endometrial adenocarcinomas, the results of this study suggest that the use of a number of markers appear useful in distinguishing the two tumours in HPV negative cases. In a clinical setting, one could use a combination of p16, CEA and vimentin in cervical tumours to determine possible site of origin. Another relevance of our results is in correct classification of cervical tumours for epidemiological research.

Our study has a number of strengths: only areas without necrosis were sampled, the use of tissue microarray techniques allowed uniform conditions during immunohistochemical staining, making the results more comparable, and all specimens were tested for HPV using sensitive methods.

Our study also has some limitations, such as the small number of samples studied, the probable variation in the fixation of the tissues due to the fact that samples came from different areas of Uganda, leading to loss of antigen epitopes. In particular, estrogen and progesterone receptors are known to be quite labile antigens.

## Conclusion

In summary, our results showed overlap in expression of p16, CEA and vimentin between HPV positive and HPV negative cervical ADC, and suggest that some samples among the HPV negative ADC, diagnosed as cervical ADC, may be of endometrial origin. In our series amongst 13 HPV negative samples, the endocervical origin seems probable in five CEA positive samples while two vimentin positive samples are suspected to be endometrial cancer. Although the number of samples studied are too few to draw definite conclusions, it suggests that CEA and vimentin may be of value in distinguishing HPV negative cervical tissue from endometrial ADC.

## List of abbreviations

ADC: adenocarcinoma; CEA: carcinoembryonic antigen; ER: estrogen receptor; HIER: Heat Induced Epitope Retrieval; HPV: human papillomavirus; IHC: immunohistochemistry; NOS: not otherwise specified; p16: cyclin dependend kinase inhibitor 2A: tumour suppressor protein; PR: progestin receptor; PRC: polymerase chain reaction; SCC: squamous cell carcinoma; TMA: tissue microarrays.

## Competing interests

The authors declare that they have no competing interests.

## Authors' contributions

EW designed the study. MO had responsibility of collection of samples and designing of the database. NG conducted laboratory analysis, while BL contributed to the project with pathological expertise. MO and EW drafted the manuscript, and all authors revised the following versions of the manuscript. All authors read and approved the final manuscript.
